# The effect of dairy consumption on the prevention of cardiovascular diseases: A meta-analysis of prospective studies

**DOI:** 10.15171/jcvtr.2017.01

**Published:** 2017-03-18

**Authors:** Fatemeh Gholami, Malihe Khoramdad, Nader Esmailnasab, Ghobad Moradi, Bijan Nouri, Saeid Safiri, Yousef Alimohamadi

**Affiliations:** ^1^Department of Epidemiology, School of Public Health, Shahid Beheshti University of Medical Sciences, Tehran, Iran; ^2^Faculty of Health, Kermanshah University of Medical Sciences, Kermanshah, Iran; ^3^Social Determinants of Health Research Center, Department of Epidemiology and Biostatistics, School of Medicine, Kurdistan University of Medical Sciences, Sanandaj, Iran; ^4^Department of Public Health, School of Public Health, Maragheh University of Medical Sciences, Maragheh, Iran; ^5^Noor Research Center for Ophthalmic Epidemiology, Noor Eye Hospital, Tehran, Iran; ^6^Department of Epidemiology, School of Public Health, Tehran University of Medical Sciences, Tehran, Iran

**Keywords:** Dairy Foods, Cardiovascular Diseases, Meta-analysis, Prospective Cohort Studies

## Abstract

***Introduction:*** There is no global consensus on the relationship of dairy products with cardiovascular diseases. This study was conducted to evaluate the effect of the consumption of dairy products on cardiovascular diseases, including stroke and coronary heart disease (CHD).

***Methods:*** Important electronic databases such as the Scopus, Science Direct, and PubMed were evaluated up to September 2014. All prospective cohort studies that evaluated the relationship between dairy products consumption and cardiovascular diseases were included regardless of their publication date and language. The study participants were evaluated regardless of age, sex, and ethnicity. The STROBE checklist was used to assess quality of the study. Two investigators separately selected the studies and extracted the data. The designated effects were risk ratio (RR) and hazard ratio (HR). The random effect model was used to combine the results.

***Results:*** Meta-analysis was performed on 27 studies. There were 8648 cases of cardiovascular diseases (CVD), 11806 cases of CHD, and 29300 cases of stroke. An inverse association was found between total dairy intake and CVD (RR=0.90, 95% CI: 0.81-0.99) and stroke (RR=0.88, 95% CI: 0.82-0.95) while no association was observed between total dairy intake and CHD. The total diary intake was associated with decreased mortality of stroke (RR=0.80, 95% CI: 0.76-0.83) although it had no association with its incidence (RR=0.96, 95% CI: 0.88-1.04).

***Conclusion:*** This is the first meta-analysis of the relationship of total dairy intake with CVD. This study showed an inverse relationship between total dairy intake and CVD while no relationship was found for CHD. Considering the limited number of studies in this regard, more studies are required to investigate the effect of different factors on the association of dairy intake and CVD.

## Introduction

Cardiovascular diseases (CVD) are one of the top 10 causes of mortality worldwide. According to the World Health Organization (WHO) statistics, an estimated 17.5 million people died from CVDs in 2012, representing 31% of all global deaths. Of these deaths, an estimated 7.4 million were due to coronary heart disease (CHD) and 6.7 million were due to stroke.^1^ In 2008, the European Union estimated that the direct and indirect costs related to CVD were €192 billion.^2^ CVD includes CHD and the diseases related to cerebral vessels^3^ (3) and is almost responsible for half of deaths in developed and 25% of deaths in developing countries.^4^ Despite the decrease of mortality from CVD in developed countries since 1970, its mortality is on the rise in developing countries.^5^

Different factors have been suggested as the causes of CVD, including behavioral risk factors related to unhealthy diet and obesity, lack of physical activity, excess alcohol consumption, and smoking, among which inappropriate diet is the most important.^6,7^ Dairy products are a major source of energy, protein, and calcium. The pattern of the dairy consumption is different in different parts of the world depending on cultural patterns and income. The highest consumption of dairy products is reported in North American and European countries. The consumption of milk is markedly high in the northernmost and Central European and North American countries while the consumption of cheese is high in Mediterranean countries like France, Italy, and Spain.^8-11^

Previous studies have published different reports regarding the effect of dairy consumption on CVD like stroke and CHD. Some researchers believe that increased intake of dairy products increases the risk of CVD^12-15^ while some other researchers believe that dairy products have a protective effect.^16-19^ The results of some studies have shown no relationship between the consumption of dairy products and the mortality of cardiovascular diseases.^20-22^

There is no global consensus on the relationship of dairy consumption with CVD. Since no meta-analysis has evaluated the effect of total dairy intake on CVD and the available studies have either addressed dairy consumption and stroke, or the number of the studies or their exposures are limited,^23-25^ It seems that a meta-analysis is essential to summarize the previous studies. Therefore, this study was conducted to summarize the results of the previous studies in order to reach a single conclusion.

## Materials and Methods

### Searching

Our search strategy was to use a combination of the following keywords: “dairy” OR “milk” OR “cheese” OR “butter” OR “cream” OR “yogurt” OR “yoghurt” AND “cerebrovascular disease” OR “cardiovascular diseases” OR “stroke” OR “cerebral infarction” AND “coronary heart disease” OR “myocardial infarction” OR “MI” OR “ischemic heart disease” OR “IHD” AND “mortality” OR “incidence” OR “survival”.

We searched three databases: PubMed (1945 to September 2014), Science Direct (1823 to September 2014), and Scopus (1973 to September 2014). We used the references of the studies, especially systematic review studies.

### Inclusion criteria

All cohort studies that evaluated the relationship between dairy intake and CVD were included in the study regardless of their language and publication year. Our population was all healthy individuals regardless of age, gender, and ethnicity; therefore, studies whose baseline population had diseases like diabetes or CVD were excluded from our study. We defined exposure as all dairy products and the outcome of the study was the incidence or mortality of CVD (stroke or CHD). CVD included stroke and CHD (WHO International Classification of Diseases [ICD]-10 I60-69; http://www.who.int/classifications/icd/en). In addition, it included cardiac arrest (I46), heart failure (I50), and sudden death (R69). CHD was considered as acute myocardial infarction, angina pectoris, and other ischemic heart disease (as in ICD- 10 I20-I25).

### Data collection and validity assessment

To ensure correct selection of the studies according to the inclusion criteria, two researchers (FG and MK) were responsible for selecting the studies independently. These researchers were not blind to the name of the authors and the journal and their results. When there was an opinion disagreement, decision was made after consulting with the third researcher (NS). The two researchers extracted the data from the selected studies. The extracted variables for data analysis included the name of the first author, study title, publication year, study location, age of the participants in the beginning of the study, sample volume, number of cases, follow-up duration, sex, type of dairy product, study outcome (incidence or mortality of CVD), RR (95% CI) or HR (95% CI) for the highest vs. lowest categories of dairy foods and variables adjusted in the analysis. RR was considered with greatest degree of control for potential confounders. If data were duplicated in more than one study, we included the study with the largest number of cases.

The STROBE checklist was used to evaluate the risk of bias and the quality of the studies.^26^ Two researchers (GF and MK) evaluated the quality of the studies separately. The evaluated items included (*a*) mentioning the study design accurately (here prospective), (*b*) explaining how the exposure was measured accurately (here dairy products), (*c*) explaining how the outcome was measured accurately (here incidence or mortality of CVD), (*d*) explaining the duration of data collection and follow-up, (*e*) explaining the inclusion and exclusion criteria, and (*f*) explaining how loss to follow-up was addressed.

### Measures of exposure effect and data analysis

Pooled measures were calculated as the inverse variance-weighted mean of the logarithm of RR and HR with 95% CI to assess the strength of association. As for the relationship of dairy products with CVD, if more than two products were evaluated in a study, they were first merged and one overall effect was reported for each study. The results were reported separately for men and women. Butter, cream, ice cream, high fat milk, cream cheese, and chocolate milk were considered high fat or whole dairy products. Stata version 12 was used for data analysis. The effect was reported using the random effect model. P value less than 0.05 was considered significant.

### Heterogeneity, publication bias and sensitivity analysis

Heterogeneity was evaluated quantitatively using I^2^ according to the Higgins classification in which I^2^ values of 25%, 50%, and 75% indicate low, moderate, and high heterogeneity, respectively.^27^ We used a funnel plot^28^ and the Egger test^29^ to evaluate publication bias.

Sensitivity analysis was performed with one study removed at a time,^30^ and a study is excessively influential if the significance of its “omitted” meta-analytic estimate differed relative to the overall estimate. Meta-regression was used to evaluate heterogeneity between studies.^31^

## Results

### Literature search and study characteristics

A total of 11 890 studies were found in the databases of which 275 were selected after evaluating their titles. Then, 143 studies were selected after removing duplication studies. After evaluating their abstracts, 62 articles with full texts were found. Five studies^32-37^ were excluded due to reporting the results of the populations of other studies^13,16,18,37^ and 30 studies were excluded because they did not report the data required for evaluating the relationship between dairy intake and CVD. Finally, 27 studies entered the meta-analysis. Ten studies with 12 separate results were entered into the CVD meta-analysis, covering a total of 140851 individuals and 8648 CVD cases. Seventeen studies with 21 separate results were entered into the CHD meta-analysis, including 47190 individuals and 11 806 CHD cases. Sixteen studies with 19 separate results were entered into the stroke meta-analysis, including 765 026 individuals and 29300 stroke cases. The FFQ questionnaire was used to estimate the consumption of dairy products in the 25 studies. One study used the 7-day household inventory method^12^ and another used to 3-day diet records^38^ to estimate the consumption of dairy products. The duration of follow-up was at least 10 years except for 3 studies.^39-41^ The results were presented according to sex in 5 studies.^18,38,40,42,43^ Twenty out of 27 studies were adjusted for important variables like age, sex, cigarette smoking, alcohol consumption, total energy intake, and BMI while adjustment for the above-mentioned variables was not performed in 7 studies.^12,13,17,43-46^ Twenty-one, four, and two studies were of high, moderate, and low quality, respectively (Table 1).

**Table 1:** Characteristics of studies included on dairy foods and stroke, CHD, CVD

**Author**	**Study, country**	**Age (y)**	**Subjects**	**Sex**	**Quality**	**Follow-up (y)**	**Outcome (cases)**	**Exposure**
Praagman et al^21^	The Rotterdam Study, Netherlands	≥55	4235	Both	High	17.3	Incidence Stroke (564) CHD (567)	Total dairy, Low-fat dairy, High-fat dairy, Total milk, Fermented dairy, Cheese, Yogurt.
Patterson et al^46^	The Swedish Mammography Cohort, Swedish	48-83	33636	Female	High	11.6	Incidence MI (1392)	Total dairy, Milk, Cultured milk/Yogurt, Cheese, Cream, Butter.
Dalmeijer et al^20^	EPIC-NL Study, Netherlands	49-70	33625	Both	High	13.1	Incidence Stroke (531) CHD (1648)	Total dairy intake, Milk and milk products, Fermented dairy, Cheese, High-fat dairy, Low-fat dairy.
Kondo et al^37^	NIPPON-DATA80, Japanese	≥30	4045 5198	Male Female	High	24	Mortality Stroke (417) CHD (174) CVD (893)	Milk and dairy products.
Maruyama et al^17^	JACC Study, Japanese	40-79	26598 37439	Male Female	High	12.6	Mortality Stroke (1077) CHD (479) CVD (2311)	Dairy products.
Louie et al^18^	BMES, Australia	49-97	2900	Both	Moderate	15	Mortality Stroke (176) CHD (432) CVD (548)	Total dairy, Low/reduced, fat dairy, Whole fat dairy.
Lin et al^43^	CVDFACTS, China	32-60	2061	Both	High	12	Incidence Stroke (123)	Dairy.
Ruesten et al^39^	EPIC-Potsdam Study, Germany	35-65	23531	Both	High	8	Incidence CVD (363)	Low-fat dairy, High-fat dairy, Low-fat cheese, High-fat cheese, butter.
Larsson et al^47^	The Swedish Mammography Cohort and the Cohort of Swedish Men	45-83	74961	Both	High	10.2	Incidence Stroke (4089)	Total dairy, Low-fat dairy, Full-fat dairy, Milk, Sour milk and yogurt, Cheese, Cream and crem fraiche.
Bernstein et al^15^	HFP, USA NHS, USA	40-75 30-55	43150 84010	Male Female	High	26 22	Incidence Stroke M: 2633 F: 1397	Whole-fat dairy, Low-fat dairy.
Soedamah-mothu et al^48^	The Whitehall 2 Cohort, UK	35-55	4255	Both	High	10	Incidence CHD(323)	Total dairy, High-fat dairy, Low-fat dairy, Milk, Fermented dairy, Yogurt , Cheese.
Avalos et al^42^	Rancho Bernardo, USA	50-93	751 1008	Male Female	High	16.2	Incidence CHD M: 222 F: 229	Non-fat milk, Yogurt, Ice-cream, Low-fat cheese, Cheese, Cottage cheese, Cream, Cream cheese, Whole milk, Butter, Milk chocolate.
van Aerde et al^14^	The Hoorn Study, Netherlands	50-75	1956	Both	High	12.4	Mortality CVD (116)	Total dairy, High-fat dairy, Low-fat dairy, Milk and milk products, Milk, cheese, Fermented dairy.
Sonsestedt et al^49^	Malmo Diet and Cancer Cohort, Sweden	44-74	26445	Both	High	12	Incidence Stroke (1176) Coronary Event (1344) CVD (2520)	Total dairy, Milk, Non-fermented milk, Fermented milk, Low-fat milk, High-fat milk, Cheese, Butter.
Goldbohm et al^41^	Netherlands Cohort Study, Netherlands	55-69	58279 62573	Male Female	High	10	Mortality Stroke (842) IHD (2689)	Milk products, Non-fermented, full-fat milk, Non-fermented low- fat milk, Milk Fermented, full-fat milk, Fermented low-fat, Cheese, Butter, Low-fat dairy.
Bernstein et al^19^	NHS, USA	30-55	84136	Female	High	26	Incidence CHD (3162)	High-fat dairy, Low-fat dairy.
Bonthuis et al^50^	Skin Cancer Prevention Trial, Australia	25-78	1529	Both	High	14.4	Mortality CVD (61)	Total dairy, Low-fat dairy, Full-fat dairy, Full-fat cheese, Milk, Yogurt.
Panagiotakos et al^40^	ATTICA Study, Greece	≥18	3042	Both	High	5	Incidence CVD (32)	Dairy products (low fat).
van der Pols et al^11^	The Body Orr Cohort, England and Scotland	8	4374	Both	High	65	Mortality CHD (378) Stroke (121)	Total dairy, Milk.
Larsson et al^13^	The ATBC Study, Finland	50-69	26556	Male	Moderate	13.6	Incidence Stroke (3281)	Total dairy, Low-fat dairy, Whole milk, Sour milk, Yogurt , Cheese, Cream, Ice-cream, butter.
Kelemen et al^51^	Iowa Women's Health Study	55-69	29017	Female	High	15	Mortality CHD (739)	Dairy
Elwood et al^36^	The Caerphilly Cohort Study, UK	45-59	2403	Male	Moderate	20-24	Incidence Stroke (185) IHD (493) CVD (628)	Milk
Sauvaget et al^44^	Life Span Study(LSS), Japanese	56	37130	Both	High	16	Mortality Stroke (1462)	Milk, Dairy products.
Ness et al^45^	The Collaborative Study, Scotland	35-64	5765	Male	Low	25	Mortality Stroke (196) CHD (892) CVD (1212)	Milk
Bostic et al^38^	The Iowa Women's Health Study, USA	55-69	34486	Female	High	8	Mortality IHD (387)	Total dairy
Kinjo et al^16^	Japanese	40-69	223170	Both	Low	15	Mortality Stroke (11030)	Dairy milk.
Mann et al^12^	The Vegetarian Society of the United Kingdom, UK	18-79	10802	Both	Moderate	13.3	Mortality IHD(64)	Milk, Cheese.

### Quantitative data synthesis

The main results are summarized in Tables 2 and 3.

**Table 2:** Summary risk estimates of the association between dairy foods and risk of CHD

	**N** ^b^	**No. of cases**	**Risk estimate (95% CI)**	**Heterogeneity test**			**References**
			**REM**	**I** ^ 2 ^ **(%)**	***P***	***P*** ^a^	
Total dairy	21	11806	0.99 (0.92-1.06)	51.6	0.003		
Outcome						0.44	
Incidence	9	7787	1.03(0.88-1.21)	44.9	0.06		[19-21, 36, 42, 46, 48, 49]
Mortality	12	4019	0.97(0.97-1.04)	51.6	0.003		[11, 12, 17, 18, 37, 38, 41, 45, 51]
**Location where the study was conducted**							
Europe	12	6136	0.98(0.89-1.08)	56.8	0.008	1	[11, 12, 20, 21, 36, 41, 45, 46, 48, 49, 51]
Asia	4	1494	0.98(0.68-1.42)	58.6	0.06	0.96	[17, 37]
Others	9	4176	1.01(0.9-1.14)	42.5	0.13	0.90	[18, 19, 38, 42]
Sex						0.95	
Male	6	2485	1.01(0.89-1.15)	35.6	0.17		[17, 36, 37, 41, 42, 45]
Female	8	6930	0.99(0.85-1.16)	64.9	0.006		[17, 19, 37, 38, 41, 42, 46, 51]
Quality							
High	17	11185	1(0.94-1.07)	45.4	0.02	1	[11, 17, 19-21, 37, 38, 41, 42, 46, 49, 51]
Moderate	3	425	0.95(0.53-1.68)	77.7	0.01	0.54	[12, 18, 36]
Low	1	196	0.68(0.40-1.14)	0	0.00	0.26	[45]
**Adjusting for 4 or more covariates(smoking, alcohol, total energy intake, BMI, Physical activity and ≥3 other dietary variables) (0.15)** ^a^							
Yes	16	10974	0.96(0.88-1.04)	47.2	0.01		[17, 19-21, 36-38, 41, 46, 48, 49, 51]
No	5	832	1.11(0.94-1.30)	46.3	0.11		[11, 12, 42, 45]
Fat content of dairy					0.74	
Low fat	11	8620	1.01(0.94-1.09)	62.6	0.00		[18-21, 41, 42, 46, 48, 49]
High fat	11	8620	0.98(0.94-1.01)	2.4	0.41		[18-21, 41, 42, 46, 48, 49]

BMI: body mass index, REM: random effect model. ^a^ P value for meta-regression, and location where the study was conducted (Europe as the reference) and Quality (high as the reference) and sex (male as the reference). ^b^ N: number of results; 2 separate results (male and female) were available in 4 studies.^17,37,41,42^

**Table 3:** Summary risk estimates of the association between dairy foods and risk of stroke

	**N** ^b^	**No. of cases**	**Risk estimate (95% CI)**	**Heterogeneity test**			**References**
			**REM**	**I** ^ 2 ^ **(%)**	***P***	***P*** ^a^	
Total dairy	19	29300	0.88(0.82-0.95)	63.1	0.00		
Outcome						0.00	
Incidence	8	13979	0.96(0.88-1.04)	49.7	0.05		[13, 15, 20, 21, 36, 43, 47, 49]
Mortality	11	15321	0.80(0.76-0.83)	0.00	0.66		[11, 16-18, 37, 41, 44, 45]
**Location where the study was conducted**							
Europe	10	10985	0.96(0.89-1.04)	29.8	0.17	1	[11, 13, 20, 21, 36, 41, 45, 47, 49]
Asia	7	14109	0.79(0.75-0.82)	0.00	0.73	0.00	[16, 17, 37, 43, 44]
Others	2	4206	0.91(0.85-0.98)	0.00	0.68	0.57	[15, 18]
Sex						0.90	
Male	7	9007	0.92(0.79-1.07)	68.5	0.00		[13, 15, 17, 36, 37, 41, 45]
Female	4	1021	0.78(0.66-0.91)	0.00	0.67		[17, 37, 41]
Quality							
High	13	10343	0.89(0.85-0.94)	0.00	0.44	1	[11, 15, 17, 20, 21, 37, 41, 43, 44, 49]
Moderate	4	7731	1.01(0.84-1.21)	63.9	0.04	0.05	[13, 18, 36, 47]
Low	2	11226	0.79(0.75-0.83)	0.00	0.90	0.26	[16, 45]
**Adjusting for 4 or more covariates (smoking, alcohol, total energy intake, BMI, physical activity and ≥3 other dietary variables) (0.10)** ^a^							
Yes	14	16368	0.91(0.85-0.98)	49.1	0.02		[13, 15, 17, 18, 20, 36, 37, 41, 47, 49]
No	5	12932	0.79(0.75-0.83)	0.00	0.98		[11, 16, 43-45]
Fat content of dairy					0.62	
Low fat	9	14689	0.94(0.90-0.98)	0.00	0.61		[13, 15, 18, 20, 41, 47, 49]
High fat	9	14689	0.95(0.91-1)	0.00	0.61		[13, 15, 18, 20, 41, 47, 49]

BMI: body mass index, REM: random effect model. ^a^ P value for meta-regression, and location where the study was conducted (Europe as the reference) and Quality (high as the reference) and sex (male as the reference). ^b^ N: number of results; 2 separate results (male and female) were available in 3 studies.^17,37,41^

### Effects of total dairy on the risk of CHD, stroke and CVD

**Figure 1:**
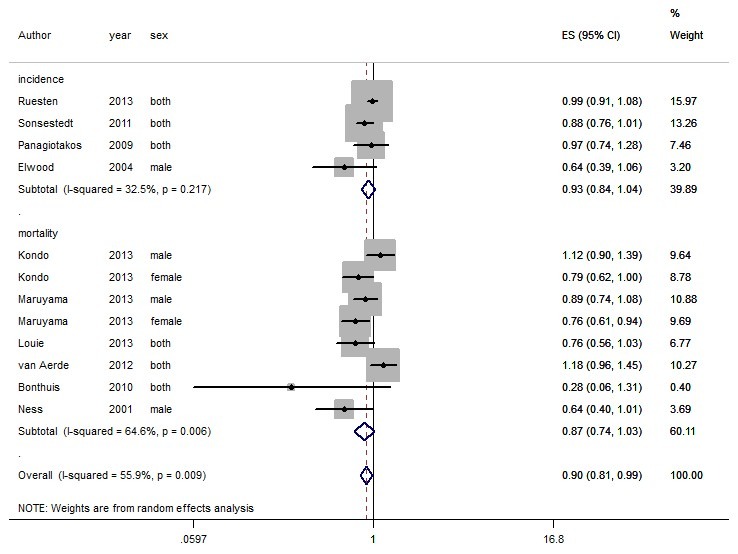
The association of total dairy foods with total CVD events, CVD incidence and CVD mortality. The size of gray box is positively proportional to the weight assigned to each study, and horizontal lines represent the 95% confidence intervals.

**Figure 2:**
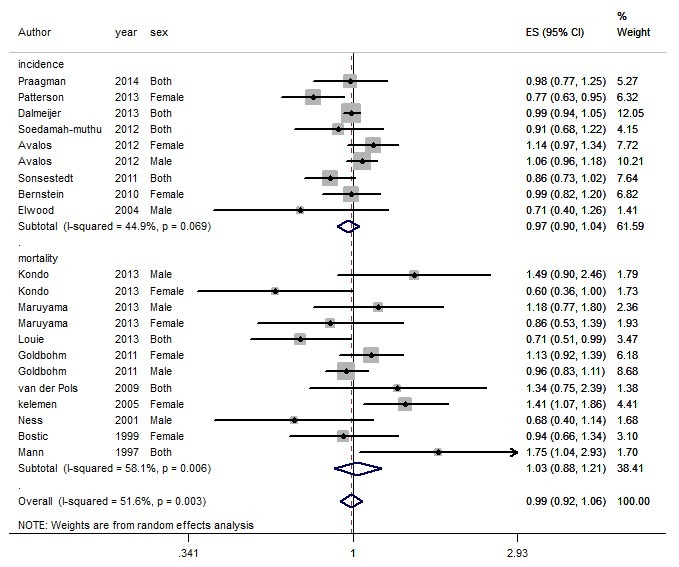
The association of total dairy foods with total CHD events, CHD incidence and CHD mortality. The size of gray box is positively proportional to the weight assigned to each study, and horizontal lines represent the 95% confidence intervals.

**Figure 3:**
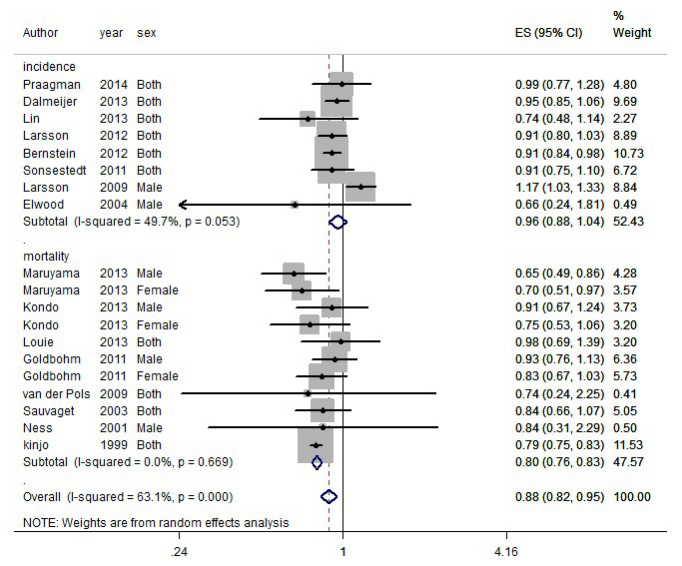
The association of total dairy foods with total stroke events, stroke incidence and stroke mortality. The size of gray box is positively proportional to the weight assigned to each study, and horizontal lines represent the 95% confidence intervals.

In general, total dairy intake reduced the risk of CVD by 10% (RR=0.90, 95% CI: 0.81-0.99) (Figure 1) while no association was observed for CHD (RR=0.99, 95% CI: 0.92-1.06) (Figure 2). Dairy intake had a protective role against stroke (RR=0.88, 95% CI: 0.82-0.95) (Figure 3).

### Heterogeneity, publication bias and sensitivity analysis

**Figure 4:**
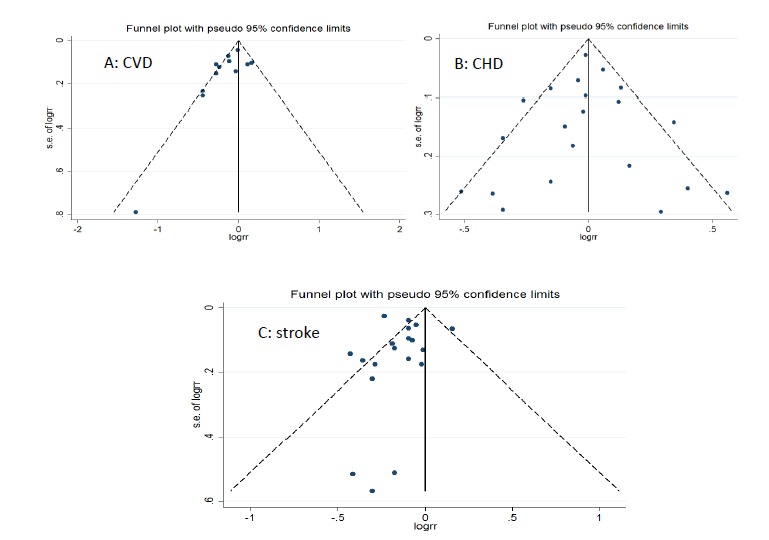
Funnel plots for the analysis of total dairy foods and CVD, CHD and stroke.

Heterogeneity was I^2^=55.8% for CVD (Figure 1). Considering the limited number of studies, it was not possible to perform meta-regression. There was publication bias according to the Egger test (*P*=0.04). Asymmetry was observed in the funnel plot (Figure 4A) due to small-study effects^47^ in a study by Bonthuis et al^48^; no change was observed in overall estimate (RR) of the study after it was excluded. The results of sensitivity analysis for CVD showed that excluding each study did not change the overall estimate of the study significantly; this relationship was 0.90, ranging from 0.78 to 1.01.

Heterogeneity was I^2^=51.6% for CHD (*P*<0.001) (Figure 2). Meta-regression was performed in the subgroups to detect the source of heterogeneity. The estimated effect had no relationship with outcome (CHD incidence: RR=0.97 [95% CI: 0.90-1.04]; CHD mortality: RR=1.03 [95% CI: 0.88-1.21]) (*P*=0.44), location (Europe: RR=0.98 [95% CI: 0.89-1.08]; Asia: RR=0.98 [95% CI: 0.68-1.42]) )*P*=0.96(, quality (high: RR=1 [95% CI: 0.94-1.07]; moderate: RR=0.95 [95% CI: 0.53-1.68]) (*P*=0.54) and low quality: (RR=0.68 [95% CI: 0.40-1.14]) (P=0.26), adjustment for 4 or more of the following covariates (smoking, alcohol, total energy intake, BMI, physical activity and ≥3 other dietary variables) yes: RR=0.96 (95% CI: 0.88-1.04); no: RR=1.11 (95% CI: 0.94-1.30) (*P*=0.15), sex/male: RR=1.01 (95% CI: 0.89-1.15); female: RR=0.99 (95% CI: 0.85-1.16) (P=0.95), and also fat content of dairy products (high-fat: RR=0.98 [95% CI: 0.94-1.01]; low-fat: RR=1.01 [95% CI: 0.94-1.09]) (*P*=0.74; Table 2).

Duration of follow-up (year) (*P*=0.71), mean age (*P*=0.91), and number of cases (*P*=0.52) were not among the sources of heterogeneity. There was no publication bias according to the Egger test (*P*=0.84). Its funnel plot is shown in Figure 4B. The results of sensitivity analysis showed that excluding each study did not change the overall estimate of the study significantly; the relationship was 0.90, ranging from 0.90 to 1.08.

Heterogeneity was I^2^=63.1% for stroke (*P*<0.001) (Figure 3). Heterogeneity changed to I^2^=34.6% after excluding the ATBC study^14^ that included male smokers, indicating that this study played an important role in heterogeneity. Meta-regression was used to detect heterogeneity. An inverse association was found between the total dairy intake and the mortality of stroke (RR=0.80 [95% CI: 0.76-0.83]) while there was no association with its incidence (RR=0.96 [95% CI: 0.88-1.04]) (*P*<0.001). Total dairy intake may lower the risk of stroke in Asian studies (RR=0.79 [95% CI: 0.75-0.82]) compared to European studies (RR=0.96 [95% CI: 0.89-1.04]) (*P*<0.001). The estimated effect had no relationship with the following: moderate quality (RR=1.01 [95% CI: 0.84-1.04]) compared to high quality (RR=0.89 [95% CI: 0.85-0.94]) (*P*=0.05) and low quality (RR=0.79 [95% CI: 0.75-0.83]) compared to high quality (*P*=0.26), adjustment for 4 or more of the following covariates (smoking, alcohol, total energy intake, BMI, physical activity and ≥ 3 other dietary variables) yes: RR=0.91 (95% CI: 0.85-0.98); no: RR=0.79 (95%CI: 0.75-0.83) (P=0.1), sex/female: RR=0.78 (95% CI: 0.66-0.91), male: RR=0.92 (95% CI: 0.79-1.07) (P=0.90), and also fat content of dairy products (high fat: RR=0.95 [95% CI: 0.91-1]; low fat: RR=0.94 [95% CI: 0.90-0.98]) (*P*=0.62) (Table 3). Duration of follow-up (year) (*P*=0.42), mean age (*P*=0.17), and number of cases (*P*=0.76) were not among the sources of heterogeneity. Although there was no publication bias according to the Egger test (*P* = 0.70), asymmetry was observed in the funnel plot (Figure 4C) due to the small-study effects of 3 studies^12,37,46^ and the overall estimate of the study effect did not change after their exclusion. The results of sensitivity analysis showed that the study estimate was 0.88, ranging from 0.81 to 0.97. After excluding the ATBC study,^14^ the study effect was 0.86, ranging from 0.81 to 0.92.

## Discussion

The findings of our meta-analysis showed that total dairy intake can lower the risk of CVD by 10% while it has no relationship with CHD. Total dairy intake has an inverse association with stroke and lowers its risk by up to 12%; this protective relationship can be attributed to low-fat dairy products while high-fat dairy products play no role in this regard. It should be noted that according to the results of our study, the consumption of dairy products only postpone stroke and lower its risk by 20% while it has no effect on its incidence.

A meta-analysis by Elwood et al in 2010^54^ investigated the relationship between dairy intake and the risk of stroke and IHD (RR=0.79, 95% CI: 0.68-0.91 and RR=0.92, 95% CI: 0.80- 0.99, respectively) and concluded that the dairy intake lowered the risk of IHD and stroke; the results of this study regarding stroke are in line with our findings while the results of IHD are slightly different due to the smaller number of IHD cases and exposure to other item like calcium intake in addition to dairy products in the study by Elwood et al.

Another meta-analysis study by Hu et al showed that the relationship between total dairy intake and stroke was protective (RR=0.88, 95% CI: 0.82-.94). This relationship was attributed to low-fat dairy products. The results of this study are in line with our findings.^23^ Moreover, in 2012, Larsson et al conducted a cohort study on a population of 74961 participants and reported that the consumption of low-fat dairy products decreased the relative risk of stroke (RR=0.88, 0.95% CI: 0.87-0.97).^55^ As for the mechanisms of this protective relationship, it can be stated that the intake of dairy products can lower the blood pressure which is an important risk factor of cardiovascular diseases.^56-58^ A study in 2009 confirmed this finding and reported that the HR of the participants who consumed low-fat dairy products was in the highest quantile (HR=0.69, 0.95% CI: 0.56-0.86).^59^

The results of a randomized controlled trial study showed that the systolic blood pressure of the individuals who used milk protein supplements was 2.3 mm Hg lower than the systolic blood pressure of those who used carbohydrates.^60^ It seems that this phenomenon is due to calcium in dairy products because calcium can decrease platelet aggregation and total cholesterol through creating insoluble complexes with fatty acids and decreasing their absorbance.^61^ Similarly, Hiroyasu reported that the risk of stroke was 31% less in women in the highest quintile of calcium intake versus those in the lowest quintile.^62^ However, it should also be noted that people who consume dairy products often pay more attention to other aspects of health which can affect their health status. For example, in 2008, a preventive medicine specialist reported that people who consumed low-fat dairy products were less likely to smoke or drink alcohol and had more physical activity and more vitamin use; moreover, they consumed more fruits, vegetables, and cereal and less red meat.^63^

A meta-analysis performed by Qin et al on the relationship of dairy foods with CVD, stroke, and CHD showed that dairy products lowered the risk of CVD by 12% which is in line with our results. However, few studies were included in this study, and combining of all dairy products was not done to investigate the relationship and limited exposures were considered. Nonetheless, this study showed the protective role of dairy intake against stroke which is similar to our findings. No relationship was found between dairy intake and CHD in this study, which is again in line with our results.^25^

A prospective study on 4000 adults in London did not show any relationship between CHD and dairy products^51^ while in the study by Elwood et al, the risk of IHD was RR=0.79 (0.95% CI: 0.68–0.91) in individuals who had the highest consumption of dairy products.^54^ It seems that the contradictory results are due to different designs, different characteristics of the participants, adjustment for different confounders, different sample sizes, and the residual confounding effects of unknown variables.

The relationship between total dairy intake and stroke was significant in Asia and not in Europe, may be due to different patterns of dairy consumption in different parts of the world. The mean dairy intake was more than 200 g/d in the United States in 2000-2005 but less than 27 g in a country like China.^8^ Another reason could be different classifications in different continents; the highest quintile was 132.6 g/d for men and 168.3 g/d for women in a study by Kondo et al in Japan in 2013^38^ while the highest quintile was 1296 g/d in the study by Larsson et al in Sweden in 2009.^14^

This study had some potential limitations and biases. It should be noted that although cohort studies are valuable ones, they are weaker than RCT studies for detecting causality; therefore, causal interpretations should be made with caution. Moreover, the limited number of studies and non-availability of the full text of some articles despite a great deal of efforts were other limitations of the study, which might have led to selection bias. On the other hand, it was not possible to calculate the crude effect because the required data were not available and analysis was performed on adjusted effects. Therefore, there is the possibility of residual confounding. However, considering the relatively good data of the studies, analysis was performed in different subgroups separately. Since few studies have evaluated the relationship between dairy consumption and different types of stroke and CHD, more studies are required in this regard. Despite the heterogeneity and lack of publication bias in our meta-analysis due to the limited number of included studies, caution should be exercised when interpreting the results. The results of sensitivity meta-analysis in stroke showed that the study by Larsson et al in 2009 was a source of heterogeneity because male smokers were investigated in this study and smoking is a risk factor of stroke.^14^ Therefore, more studies are required to shed light on the relationship of dairy intake in male smokers and stroke. Considering these limitations, more studies should be performed in this regard. However, our study showed the protective role of dairy consumption on stroke and CVD which is of clinical significance.

## Conclusion

This is the first meta-analysis addressing the relationship between total dairy intake and CVD. The results showed a possible inverse association between total dairy intake and CVD and stroke while no association was observed between total dairy intake and CHD. Due to the limited number of studies in this regard, more studies investigating all factors associated with cardiovascular diseases are required to make a definite conclusion.

## Ethical Approval

The local ethical committee reviewed and approved the study.

## Competing interests

None.

## Acknowledgements

We would like to thank all those researchers who helped us to conduct this study.

## References

[1] WHO. Cardiovascular diseases: Fact sheet No. 317. September 2012. Available from http://www.who.int/mediacentre/factsheets/fs317/en/.

[2] Allender S, Scarborough P, Peto V, Rayner M, Leal J, Luengo-Fernandez R, et al. European Cardiovascular Disease Statistics. Oxford: European Heart Network, 2008.

[3] Mendis S, Puska P, Norrving B. Global atlas on cardiovascular disease prevention and control. Geneva: World Health Organization; 2011‏.

[4] Adnan Y, Qureshi Ms, Fawad A, Gul AM, Hafizullah M (2013). Frequency Of cardiovascular risk factors among dairy workers. Pakistan Heart Journal.

[5] Sarraf-Zadegan N, Boshtam M, Malekafzali H, Bashardoost N, Sayed-Tabatabaei F, Rafiei M (1999). Secular trends in cardiovascular mortality in Iran, with special reference to Isfahan. Acta Cardiologica.

[6] WHO. Media centre: cardiovascular diseases (CVDs). 2011‏.

[7] Esmailnasab N, Moradi G, Delaveri A (2012). Risk factors of non-communicable diseases and metabolic syndrome. Iran J Public Health.

[8] Wang Y, Li S (2008). Worldwide trends in dairy production and consumption and calcium intake: is promoting consumption of dairy products a sustainable solution for inadequate calcium intake?. Food Nutr Bull.

[9] Wahlqvist ML (2007). Regional food culture and development. Asia Pac J Clin Nutr.

[10] Sanchez-Villegas A, Martinez J, Prättälä R, Toledo E, Roos G, Martinez-Gonzalez M (2003). A systematic review of socioeconomic differences in food habits in Europe: consumption of cheese and milk. Eur J Clin Nutr.

[11] Artaud-Wild SM, Connor S, Sexton G, Connor W (1993). Differences in coronary mortality can be explained by differences in cholesterol and saturated fat intakes in 40 countries but not in France and Finland A paradox. Circulation.

[12] Van der Pols J, Gunnell D, Williams G, Holly J, Bain C, Martin R (2009). Childhood dairy and calcium intake and cardiovascular mortality in adulthood: 65-year follow-up of the Boyd Orr cohort. Heart.

[13] Mann JI, Appleby PN, Key TJ, Thorogood M (1997). Dietary determinants of ischaemic heart disease in health conscious individuals. Heart.

[14] Larsson SC, Männistö S, Virtanen MJ, Kontto J, Albanes D, Virtamo J (2009). Dairy foods and risk of stroke. Epidemiology (Cambridge, Mass).

[15] van Aerde MA, Soedamah-Muthu SS, Geleijnse JM, Snijder MB, Nijpels G, Stehouwer CD (2013). Dairy intake in relation to‏ cardiovascular disease mortality and all-cause mortality: the Hoorn Study. Eur J Nutr.

[16] Bernstein AM, Pan A, Rexrode KM, Stampfer M, Hu FB, Mozaffarian D (2012). Dietary protein sources and the risk of stroke in men and women. Stroke.

[17] Kinjo Y, Beral V, Akiba S, Key T, Mizuno S, Appleby P (1999). Possible protective effect of milk, meat and fish for cerebrovascular disease mortality in Japan. J Epidemiol.

[18] Maruyama K, Iso H, Date C, Kikuchi S, Watanabe Y, Wada Y (2013). Dietary patterns and risk of cardiovascular deaths among middle-aged Japanese: JACC Study. Nutr Metab Cardiovasc Dis.

[19] Louie JCY, Flood VM, Burlutsky G‏, Rangan AM, Gill TP, Mitchell P (2013). Dairy consumption and the risk of 15-year cardiovascular disease mortality in a cohort of older Australians. Nutrients.

[20] Bernstein AM, Sun Q, Hu FB, Stampfer MJ, Manson JE, Willett WC (2010). Major dietary protein sources and risk of coronary heart disease in women. Circulation.

[21] Dalmeijer GW, Struijk EA, van der Schouw YT, Soedamah-Muthu SS, Verschuren WM, Boer JM (2013). Dairy intake and coronary heart disease or stroke—a population-based cohort study. Int J Cardiol.

[22] Praagman J, Franco OH, Ikram MA, Soedamah-Muthu SS, Engberink MF, van Rooij FJ (2015). Dairy products and the risk of stroke and coronary heart disease: the Rotterdam Study. Eur J Nutr.

[23] Hu D, Huang J, Wang Y, Zhang D, Qu Y (2014). Dairy foods and risk of stroke: a meta-analysis of prospective cohort studies. Nutr Metab Cardiovasc Dis.

[24] Soedamah-Muthu SS‏, Ding EL, Al-Delaimy WK, Hu FB, Engberink MF, Willett WC (2011). Milk and dairy consumption and incidence of cardiovascular diseases and all-cause mortality: dose-response meta-analysis of prospective cohort studies. Am J Clin Nutr.

[25] Qin L-Q, Xu J-Y, Han S-F, Zhang Z-L, Zhao Y-Y, Szeto IM (2015). Dairy consumption and risk of cardiovascular disease: an updated meta-analysis of prospective cohort studies. Asia Pac J Clin Nutr.

[26] Von Elm E, Altman DG, Egger M, Pocock SJ, Gøtzsche PC, Vandenbroucke JP (2008). The Strengthening the Reporting of Observational Studies in Epidemiology [STROBE] statement: guidelines for reporting observational studies. Gaceta Sanitaria.

[27] Huedo-Medina TB, Sánchez-Meca J, Marín-Martínez F, Botella J (2006). Assessing heterogeneity in meta-analysis: Q statistic or I² index?. Psychol Methods.

[28] Higgins JP, Green S. Cochrane handbook for systematic reviews of interventions: Wiley Online Library; 2008‏.

[29] Egger M, Smith GD, Schneider M, Minder C (1997). Bias in meta-analysis detected by a simple, graphical test. BMJ.

[30] Tobias A (1999). Assessing the influence of a single study in the meta-anyalysis estimate. Stata Technical Bulletin.

[31] Higgins J, Thompson SG (2004). Controlling the risk of spurious findings from meta‐regression. Stat Med.

[32] He K, Merchant A, Rimm EB, Rosner BA, Stampfer MJ, Willett WC (2003). Dietary fat intake and risk of stroke in male US healthcare professionals: 14 year prospective cohort study. BMJ.

[33] Hu FB, Stampfer MJ, Manson JE, Ascherio A, Colditz GA, Speizer FE (1999). Dietary saturated fats and their food sources in relation to the‏ risk of coronary heart disease in women. Am J Clin Nutr.

[34] Appleby PN, Thorogood M, Mann JI, Key TJ (1999). The Oxford vegetarian study: an overview. Am J Clin Nutr.

[35] Elwood PC, Strain J, Robson PJ, Fehily AM, Hughes J, Pickering J (2005). Milk consumption, stroke, and heart attack risk: evidence from the Caerphilly cohort of older men. J Epidemiol Community Health.

[36] Eguchi E, Iso H, Tanabe N, Wada Y, Yatsuya H, Kikuchi S (2012). Healthy lifestyle behaviours and cardiovascular mortality among Japanese men and women: the Japan collaborative cohort study. Eur Heart J.

[37] Elwood PC, Pickering JE, Fehily‏ A, Hughes J, Ness A (2004). Milk drinking, ischaemic heart disease and ischaemic stroke I Evidence from the Caerphilly cohort. Eur J Clin Nutr.

[38] Kondo I, Ojima T, Nakamura M, Hayasaka S, Hozawa A, Saitoh S (2013). Consumption of dairy products and death from cardiovascular disease in the Japanese general population: the NIPPON DATA80. J Epidemiol.

[39] Bostick RM, Kushi LH, Wu Y, Meyer KA, Sellers TA, Folsom AR (1999). Relation of calcium, vitamin‏ D, and dairy food intake to ischemic heart disease mortality among postmenopausal women. Am J Epidemiol.

[40] Von Ruesten A, Feller S, Bergmann M, Boeing H (2013). Diet and risk of chronic diseases: results from the first 8 years of follow-up in the EPIC-Potsdam study. Eur J Clin Nutr.

[41] Panagiotakos D, Pitsavos C, Chrysohoou C, Palliou K, Lentzas I, Skoumas I (2009). Dietary patterns and 5-year incidence of cardiovascular disease: a‏ multivariate analysis of the ATTICA study. Nutr Metab Cardiovasc Dis.

[42] Goldbohm RA, Chorus AM, Garre FG, Schouten LJ, van den Brandt PA (2011). Dairy consumption and 10-y total and cardiovascular mortality: a prospective cohort study in the Netherlands. Am J Clin Nutr.

[43] Avalos EE, Barrett-Connor E, Kritz-Silverstein D, Wingard DL, Bergstrom JN, Al-Delaimy WK (2013). Is dairy product consumption associated with the incidence‏ of CHD?. Public Health Nutr.

[44] Lin P-H, Yeh W-T, Svetkey LP, Chuang S-Y, Chang Y-C, Wang C (2013). Dietary intakes consistent with the DASH dietary pattern reduce blood pressure increase with age and risk for stroke in a Chinese population. Asia Pac J Clin Nutr.

[45] Sauvaget C, Nagano J, Allen N, Grant EJ, Beral V (2003). Intake of animal products and stroke mortality in the Hiroshima/Nagasaki Life Span Study. Int J Epidemiol.

[46] Ness A, Smith GD, Hart C (2001). Milk, coronary heart disease and mortality. J Epidemiol Community Health.

[47] Sterne JA, Gavaghan D, Egger M (2000). Publication and related bias in meta-analysis: power of statistical tests and prevalence in the literature. Journal of Clinical Epidemiology.

[48] Bonthuis M, Hughes M, Ibiebele T, Green A, Van Der Pols (2010). Dairy consumption and patterns of mortality of Australian
adults. Eur J Clin Nutr.

[49] Praagman J, Franco OH, Ikram MA, Soedamah-Muthu SS, Engberink MF, van Rooij FJ (2015). Dairy products and the risk of stroke and coronary heart disease: the Rotterdam Study. Eur J Nutr.

[50] Patterson E, Larsson SC, Wolk A, Åkesson A (2013). Association between dairy food consumption and risk of myocardial infarction in women differs by type of dairy food. J Nutr.

[51] Soedamah-Muthu SS‏, Masset G, Verberne L, Geleijnse JM, Brunner EJ (2013). Consumption of dairy products and associations with incident diabetes, CHD and mortality in the Whitehall II study. Br J Nutr.

[52] Sonestedt E, Wirfält E, Wallström P, Gullberg B, Orho-Melander M, Hedblad B (2011). Dairy products and its association with incidence of cardiovascular disease: the Malmö diet and cancer cohort. Eur J Epidemiol.

[53] Kelemen LE, Kushi LH, Jacobs DR, Cerhan JR (2005). Associations of dietary protein with disease and mortality in a prospective study of postmenopausal women. Am J Epidemiol.

[54] Elwood PC, Pickering JE, Givens DI, Gallacher JE (2010). The consumption of milk and dairy foods and the incidence of vascular disease and diabetes: an overview of the evidence. Lipids.

[55] Larsson SC, Virtamo J, Wolk A (2012). Dairy consumption and risk of stroke in Swedish women and men. Stroke.

[56] Ackley S, Barrett-Connor E, Suarez L (1983). Dairy products, calcium, and blood pressure. Am J Clin Nutr.

[57] Benatar JR, Sidhu K, Stewart RA (2013). Effects of high and low fat dairy food on cardio-metabolic risk factors: a meta-analysis of randomized studies. PLoS One.

[58] Romaguera D, Ängquist L, Du H, Jakobsen MU, Forouhi NG, Halkjær J (2011). Food composition of the diet in relation to changes in waist circumference adjusted for body mass index. PLoS One.

[59] Engberink MF, Hendriksen MA, Schouten EG, van Rooij FJ, Hofman A, Witteman JC (2009). Inverse association between dairy intake and hypertension: the Rotterdam Study. Am J Clin Nutr.

[60] He J, Wofford MR‏, Reynolds K, Chen J, Chen C-S, Myers L (2011). Effect of dietary protein supplementation on blood pressure: a randomized, controlled trial. Circulation.

[61] Umesawa M, Iso H, Ishihara J, Saito I, Kokubo Y, Inoue M (2008). Dietary calcium‏ intake and risks of stroke, its subtypes, and coronary heart disease in Japanese: The JPHC Study Cohort I. Stroke.

[62] Iso H, Stampfer MJ, Manson JE, Rexrode K, Hennekens CH, Colditz GA (1999). Prospective study of calcium, potassium, and magnesium intake and risk of stroke in women. Stroke.

[63] Wang L, Manson JE, Buring JE, Lee I-M, Sesso HD (2008). Dietary intake of dairy products, calcium, and vitamin D and the risk of hypertension in middle-aged and older women. Hypertension.

